# Genetic features of avian influenza (A/H5N8) clade 2.3.4.4b isolated from quail in Egypt

**DOI:** 10.1016/j.virusres.2024.199482

**Published:** 2024-10-17

**Authors:** Mohamed H. Elhusseiny, Moataz M. Elsayed, Wesam H. Mady, Osama Mahana, Neveen R. Bakry, Ola Abdelaziz, Abdel-Sattar Arafa, Momtaz A. Shahein, Samah Eid, Mahmoud M. Naguib

**Affiliations:** a Reference Laboratory for Veterinary Quality Control on Poultry Production, Animal Health Research Institute, Agriculture Research Center (ARC), Giza, Egypt; b General Organization for Veterinary Services, Giza, Egypt; c Zoonosis Science Center, Department of Medical Biochemistry and Microbiology, Uppsala University, Uppsala, Sweden; d Department of Infection Biology & Microbiomes, Institute of Infection, Veterinary and Ecological Sciences, University of Liverpool, Liverpool L3 5RF, UK

**Keywords:** Avian influenza virus, Egypt, Quail, Virus evolution, H5N8

## Abstract

Several genotypes of the highly pathogenic avian influenza (HPAI) virus H5N8 subtype within clade 2.3.4.4b continue to circulate in different species of domestic birds across Egypt. It is believed that quail contribute to virus replication and adaptation to other gallinaceous poultry species and humans. This study provides genetic characterization of the full genome of HPAI H5N8 isolated from quail in Egypt. The virus was isolated from a commercial quail farm associated with respiratory signs. To characterize the genetic features of the detected virus, gene sequencing via Sanger technology and phylogenetic analysis were performed. The results revealed high nucleotide identity with the HPAI H5N8 virus from Egypt, which has multiple basic amino acid motifs PLREKRRKR/GLF at the hemagglutinin (HA) cleavage site. Phylogenetic analysis of the eight gene segments revealed that the quail isolate is grouped with HPAI H5N8 viruses of clade 2.3.4.4b and closely related to the most recent circulating H5N8 viruses in Egypt. Whole-genome characterization revealed amino acid preferences for avian receptors with few mutations, indicating their affinity for human-like receptors and increased virulence in mammals, such as S123P, S133A, T156A and A263T in the HA gene. In addition, the sequencing results revealed a lack of markers associated with influenza antiviral resistance in the neuraminidase and matrix-2 coding proteins. The results of the present study support the spread of HPAIV H5N8 to species other than chickens in Egypt. Therefore, continuous surveillance of AIV in different bird species in Egypt followed by full genomic characterization is needed for better virus control and prevention.

## Highlights

• This study reports genetic analyses of the H5N8 virus in quail in Egypt.
• The virus is phylogenetically related to other H5N8 viruses within clade 2.3.4.4b.
• These findings support the spread of the H5N8 virus to other bird species in Egypt.
• Recording mutations indicate their affinity for human-like receptors and increased virulence in mammals.
• This calls for continuous surveillance and genetic monitoring of the influenza virus.

## 1 Introduction

Avian influenza viruses (AIV) continue to cause outbreaks in several bird species and threatens public health (Krammer et al., 2018). Global outbreaks caused by the highly pathogenic avian influenza (HPAI) virus have resulted in severe economic losses to the poultry industry (Boni et al., 2013). Furthermore, the potential of HPAI viruses to spread across the species barrier and infect humans and other animals raises the possibility of a pandemic (Short et al., 2015). The AIV is an enveloped, negative-sense, eight-segment single-stranded RNA genome belonging to the family Orthomyxoviridae. The following genes are included in the eight segments: polymerase basic 1 (PB1), polymerase basic 2 (PB2), polymerase acidic (PA), hemagglutinin (HA), nucleoprotein (NP), neuraminidase (NA), matrix (M), and nonstructural (NS) genes. These genes encode 11 proteins (PB2, PB1, PB1-F2, PA, HA, NP, NA, M1, M2, NS1, and NS2) (Lamb and Choppin, 1983; Fields et al., 2007).

The HPAI H5N8 virus was first detected in Egypt in late 2016 in migratory birds (Selim et al., 2017; Kandeil et al., 2017). The virus was genetically closely related to the Russian HPAI H5N8 virus of clade 2.3.4.4b in 2016 (Yehia et al., 2018). Several genotypes of the HPAI H5N8 virus clade 2.3.4.4.b have been reported in different species of domestic birds throughout Egypt (Yehia et al., 2018; Salaheldin et al., 2018). In addition, H5N8, an HPAI H5N1 virus of clade 2.3.4.4b, was recently reported in Egypt (Mosaad et al., 2023). Moreover, the cocirculation of the Egyptian HPAI H5N8 virus with other low-pathogenicity avian influenza virus (LPAI) H9N2 strains contributed to the emergence of novel HPAI H5N2 viruses in domestic chickens and ducks (Hagag et al., 2019; Hassan et al., 2020). The circulation of several AIV subtypes in domestic birds and the emergence of novel subtypes prompted the study of AIV in mixed vessel hosts.

Quails are more susceptible to aquatic bird-derived AIVs than chickens are and are considered intermediate hosts for duck viruses among terrestrial fowl (Makarova et al., 2003; Perez et al., 2003). Since infected quail typically shed large amounts of virus without exhibiting any symptoms or mortality in certain AIV subtypes, quail may aid in virus replication and adaptation to other gallinaceous poultry species and humans. (Makarova et al., 2003; Bonfante et al., 2013; Yamada et al., 2012).

Several reports have explored the genetic characterization of HPAI viruses in chickens, turkeys, domestic ducks, and wild aquatic birds (Selim et al., 2017; Yehia et al., 2018; Hassan et al., 2020; Yehia et al., 2020; Moatasim et al., 2019), with limited data on viruses isolated from quail - one isolate with partial nucleotide sequence of its HA sequence (Shehata et al., 2019). Hence, this study aimed to characterize the genetic features of HPAI H5N8 viruses isolated from Quail in Egypt and assess their relatedness with contemporary viruses circulating in other species.

## 2 Materials and methods

Tracheal swabs were collected from a suspected commercial quail farm in Giza Governorate, Egypt, as part of passive surveillance, showing diarrhea, discharge from the nose, coughing and sneezing symptoms, and high mortality. The collected samples were analysed at the Reference Laboratory of Veterinary Quality Control on Poultry Production (RLQP). Viral RNA from pooled tracheal swabs was extracted via the QIAamp Viral RNA Mini Kit (Qiagen, Hilden, Germany) according to the manufacturer's instructions. The extracted RNA was initially tested for the matrix (M) gene of influenza A viruses via reverse transcription polymerase chain reaction (RT‒qPCR) (Spackman et al., 2003) with an AgPath-ID™ one-step RT‒PCR kit (Life Technologies, Austin, USA) and then tested via gene-specific RT‒qPCR assays for the hemagglutinin (HA) and neuraminidase (NA) gene segments of AIV H5 and N1, N2, and N8 (Hoffmann et al., 2016). RT‒qPCR was conducted with a Stratagene MX3005P real-time PCR machine (Agilent, Santa Clara, CA, USA). The positive sample was isolated via inoculation into the allantoic cavity of 9-day-old specific pathogen-free (SPF) embryonated chicken eggs (ECEs). Using 1% chicken red blood cells, a hemagglutination assay was used to detect the virus in the allantoic fluid. According to the standard protocols of the WOAH (World Organization for Animal Health) diagnostic manual (WOAH, 2021). The AIV HA and NA genes were subsequently tested via RT‒qPCR as previously described. The viruses analysed in our current study were obtained after a single passage in embryonated chicken eggs to minimize potential changes caused by adaptation to chicken eggs (Widjaja et al., 2006).

Furthermore, whole-genome sequencing was performed via a set of forward and reverse primers for each influenza A virus gene segment as described previously (Höper et al., 2009)]. Specific RT‒PCR-generated genomic segments of influenza A virus were initially subjected to size separation via agarose gel electrophoresis. The separated segments were then excised from the gel and purified via a QIAquick Gel Extraction Kit (Qiagen, Hilden, Germany). Following purification, the PCR products were directly utilized for cycle sequencing reactions using the BigDye Terminator v3.1 Cycle Sequencing Kit (Applied Biosystems, Waltham, MA, USA). The sequence-amplified products were subsequently purified via Centrisep spin columns (Thermo Fisher, Waltham, MA, USA). The purified products were then subjected to direct sequencing via an ABI PRISM 3100 Genetic Analyser (Life Technologies, Carlsbad, CA, USA). The obtained sequences were assembled via Geneious Prime 2024.0.5 (https://www.geneious.com). A BLAST search was performed via the Basic Local Alignment Search Tool (BLASTn) at NCBI https://blast.ncbi.nlm.nih.gov/Blast.cgi .

Additionally, genetic sequences of representative global HPAI H5N8 viruses from clade 2.3.4.4, as well as Egyptian HPAI H5N8 viruses, were obtained from the GISAID platform (GISAID, http://www.gisaid.org). The selection of representative viruses was based on geographical locations and the availability of whole-genome sequences to ensure a balanced representation in all eight gene segment analyses. The nucleotide sequences of both the retrieved viruses and those obtained in this study were aligned via MAFFT (Katoh and Standley, 2013). Phylogenetic trees were then constructed, employing the maximum likelihood methodology after selecting the best-fit model on the basis of the Akaike criterion via the W-IQ-TREE Web Service http://iqtree.cibiv.univie.ac.at (Nguyen et al., 2015). Finally, the phylogenetic trees were annotated and visualized via FigTree v1.4.4 software (http://tree.bio.ed.ac.uk/software/figtree/, accessed on 5 May 2024) and Inkscape 1.0 (https://inkscape.org).

## 3 Results

Swab samples obtained from the quail farm were found positive for AIV and subtypes by RT-PCR as H5N8 subtype. The virus was successfully isolated in ECEs and named A/quail/Egypt/FAO-S98/2021 (hereafter, “A/quail/FAO-S98”). Nucleotide sequences were obtained for all influenza gene segments and sequences established in this study were submitted to GenBank under accession numbers JX438661, MH893737, MF037848, KR732532, CY020649, EU146856, KR732442, and KR732472.

The hemagglutinin (HA) of the quail/FAO-S98revealed multiple basic amino acid motifs, PLREKRRKR/GLF, at the HA cleavage site, confirming its highly pathogenic status. The receptor binding pocket of the HA protein of quail/FAO-S98 displayed amino acids H103, L129, E186, G221, Q222, and G224, suggesting an avian-like α2,3-sialic acid receptor binding preference (Cai et al., 2012; Mair et al., 2014). Genetically, A/quail/FAO-S98 displayed T140A and V522A, as previously reported in Egyptian HPAI H5N8 (Hagag et al., 2022). The quail/FAO-S98strain is characterized by unique amino acid I162 M, R169Q, and I198 V substitution mutations in the HA gene compared to the consensus of other HPAI H5N8 viruses reported in Egypt. The amino acid mutations A140, M162, and Q169 present in the HA of the A/quail/FAO-S98 were not found in A/Quail/Sharkia/MEVACF25/2016, GenBank accession number MH349014. Furthermore, phenotypic markers associated with increased pathogenicity in mammals and increased virus binding to α 2,6 sialic acid receptors were detected in the sequenced quail isolate. Among 31 markers (Table 1), 29 detected markers that were previously observed in the circulating HPAI H5N8 strains since the first incursion in 2016, whereas two mutations in the NS1 and NS2 genes, D189N and M31I, respectively, are found in the A/quail/FAO-S98 and in four recent HPAI H5N8 strains from Egypt in 2021 (OL354986, OL354895, OL353693, and OL354904), according to published HPAI H5N8 strains in the GenBank. These two mutations were described to potentially increase virulence in mammals (Lycett et al., 2009; Subbarao and Shaw, 2000). Additionally, of the 29 markers, two positions in the HA revealed substitutions other than those previously reported in other studies. The first is Q192R/H (Li et al., 2009; Yen et al., 2009), which was found as 192K in the A/quail/FAO-S98, and the second is S223N (Chutinimitkul et al., 2010; Gambaryan et al., 2006); which was recorded as 223R in the A/quail/FAO-S98. No substitutional amino acid mutations related to oseltamivir (E117, Q134, G145, H273, R291, and N293) or amantadine (L26, V27, A30, S31, and G34) resistance were detected in NA or M2, respectively.

**Table 1:** Phenotypic markers reported in the quail/FAO-S98isolate analysed in this study among all coding proteins. We used the H5 numbering system to designate amino acid positions in our investigation.

Gene	Mutation	Quail	trait	References
PB2	T63I	I	Pathogenic in mice	(Li et al., 2011)
	L89V	V	Enhanced polymerase activity, Increased virulence in mice	(Li et al., 2009)
	G309D	D	Enhanced polymerase activity, Increased virulence in mice	(Li et al., 2009)
	T339K	K	Enhanced polymerase activity, Increased virulence in mice	(Li et al., 2009)
	Q368R	R	Increased polymerase activity, Increased virulence in mammals	(Salomon et al., 2006; Govorkova et al., 2005)
	H447Q	Q	Increased polymerase activity, Increased virulence in mammals	(Salomon et al., 2006; Govorkova et al., 2005)
	R477G	G	Enhanced polymerase activity, Increased virulence in mice	(Li et al., 2009)
	I495V	V	Enhanced polymerase activity, Increased virulence in mice	(Li et al., 2009)
	A676T	T	Enhanced polymerase active, Increased virulence in mice	(Li et al., 2009)
PB1	R207K	K	Increased polymerase activity in mammalian cells	(Hulse-Post et al., 2007)
	K328N	N	Increased polymerase activity, Increased virulence in mammals	(Salomon et al., 2006; Govorkova et al., 2005)
	S375N/T	N	Increased polymerase activity, Increased virulence in mammals, Human host marker	(Salomon et al., 2006; Govorkova et al., 2005; Taubenberger et al., 2005)
	H436Y	Y	Increased polymerase activity and virulence in mallards, ferrets and mice	(Hulse-Post et al., 2007)
	L473V	V	Increased polymerase activity and replication efficiency	(Xu et al., 2012)
	M677T	T	Pathogenic in mice	(Li et al., 2011)
PA	H266R	R	Increased polymerase activity, Increased virulence in mammals and birds	(Li et al., 2011)
	S/A515T	T	Increased polymerase activity, Increased virulence in mammals and birds	(Hulse-Post et al., 2007; Taubenberger et al., 2005; Xu et al., 2012; Leung et al., 2010)
HA	S123P	P	Increased virus binding to 2,6.	(Yamada et al., 2006; Watanabe et al., 2011; Chutinimitkul et al., 2010; Gambaryan et al., 2006; Li et al., 2011; Li et al., 2009; Salomon et al., 2006; Govorkova et al., 2005; Hulse-Post et al., 2007; Taubenberger et al., 2005; Xu et al., 2012; Leung et al., 2010; Yang et al., 2012)
	S133A	A	Increased pseudovirus binding to 2,6.	(Yang et al., 2007)
	T156A	A	Airborne transmissible in mammals.	(Yen et al., 2009; Herfst et al., 2012)
	Q192R/H	K	Increased virus binding to 2,6.	(Yamada et al., 2006; Watanabe et al., 2011)
	S223N	R	Increased virus binding to 2,6.	(Chutinimitkul et al., 2010; Gambaryan et al., 2006)
	A263T	T	Increased virulence in mammals.	(Lycett et al., 2009; Wu et al., 2008)
NA	T223I	I	Increased virulence in mammals	(Chen et al., 2007; Lee et al., 2007; Katz et al., 2000)
M1	N30D	D	Increased virulence in mammals	(Fan et al., 2009)
	T215A	A	Increased virulence in mammals	(Fan et al., 2009)
NS1	A/P42S	S	Increased virulence in mammals, Antagonism of IFN induction	(Lycett et al., 2009; Jiao et al., 2008)
	T/D/V/R/A127N	N	Increased virulence in mammals	(Lycett et al., 2009; Min et al., 2007)
	V149A	A	Pathogenicity in mice, Antagonism of IFN induction	(Li et al., 2006)
	D/G189N	N	Increased virulence in mammals	(Lycett et al., 2009; Subbarao and Shaw, 2000)
NS2	M31I	I	Increased virulence in mammals	(Lycett et al., 2009; Subbarao and Shaw, 2000)

**Fig. 1:**
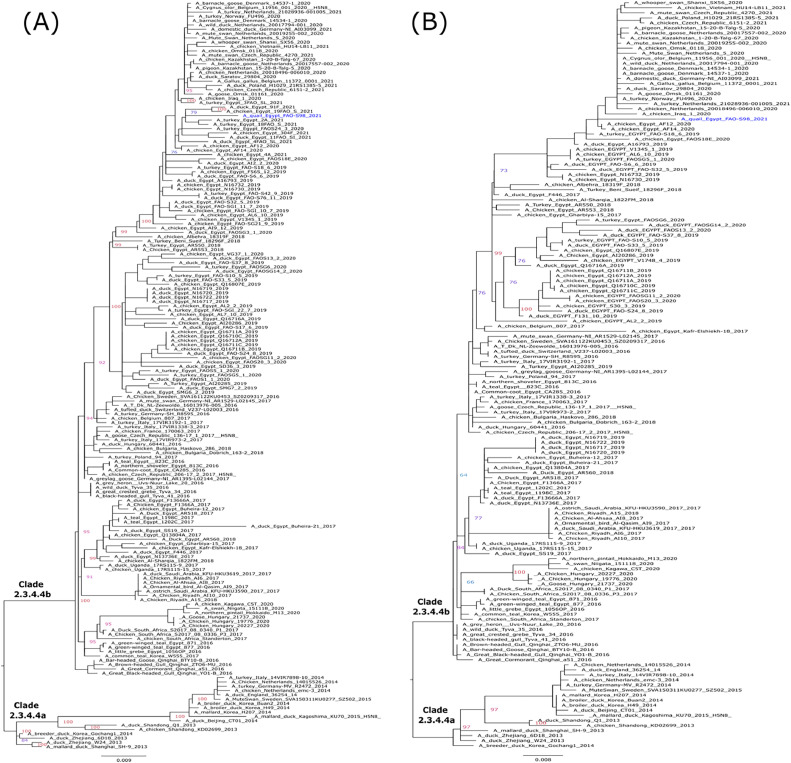
Phylogenetic relationships of the HA (a) NA (b) gene segments of Egyptian and representative H5N8 viruses. Egyptian H5N8 virus from Quail is colored in blue. Trees were generated, after the selection of the best-fitted model (HA: GTR+*F* + *I* + G4, NA:TVM+*F* + G4), by employing maximum likelihood methodology based on Bayesian information criterion using IQ-tree software version 1.1.3 (Katoh and Standley, 2013).

**Fig. 2:**
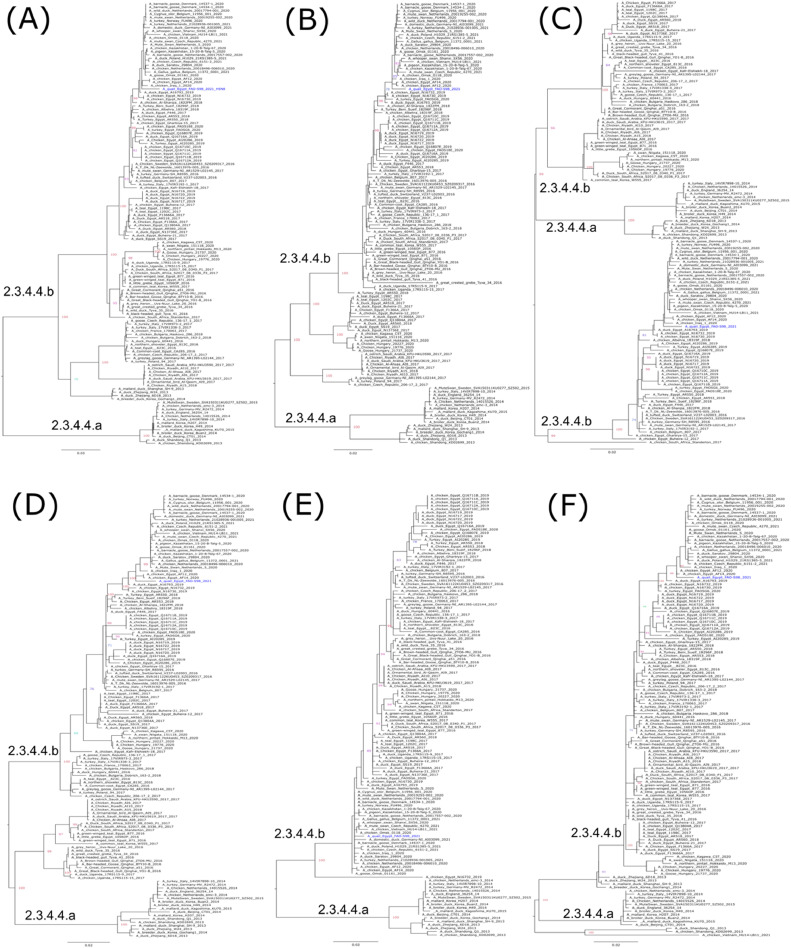
Phylogenetic relationships of the PB2 (A), PB1 (B), PA (C), NP (D), M (E), and NS (F) gene segments of Egyptian and representative H5N8 viruses. Egyptian H5N8 virus from Quail is colored in blue. Trees were generated, after the selection of the best-fitted model (PB2: GTR+*F* + G4, PB1:GTR+*F* + G4, PA:TVM+*F* + G4, NP:TPM2u+*F* + G4, M:TIM2e+G4, and NS: K3Pu+*F* + G4), by employing maximum likelihood methodology based on Bayesian information criterion using IQ-tree software version 1.1.3.

Phylogenetic analyses match the genetic characterization and reveal that the HA and NA gene segments of the A/quail/FAO-S98 H5N8 virus are closely related to other Egyptian HPAI H5N8 viruses of clade 2.3.4.4b. The topology of the trees revealed a phylogenetic relatedness of the A/quail/FAO-S98 virus with HPAI H5N8 viruses circulating in Egypt 2020–2021 and HPAI H5N8 viruses from Europe 2020–2021 in all eight gene segments (Figs. 1a, b and 2a–f).

## 4 Discussion

The HPAI H5N8 virus was first detected in Egypt in late 2016 in migrating birds (Selim et al., 2017; Kandeil et al., 2017). Later, based on surveillance and the whole-genome sequence of viruses from different governorates, approximately six genotypes of the HPAI H5N8 virus were characterized in Egypt's migratory and domestic bird populations (Yehia et al., 2018; Salaheldin et al., 2018). The HPAI H5N8 virus has contributed to the emergence of novel HPAI H5N2 viruses in Egypt (Hagag et al., 2019; Hassan et al., 2020). This emphasizes how crucial it is to track the molecular characterization of HPAI H5Nx strains that are now spreading among various bird species, including uncommon ones such as quail. The current study aimed to genetically characterize the full genome sequence of the HPAI H5N8 virus isolated from quail farms in Egypt.

The molecular characterization of the A/quail/FAO-S98 revealed that the HA cleavage site was polybasic in nature and harbored an amino acid sequence (PLREKRRKR/GLF), indicating a highly pathogenic phenotype that matches the most recent circulating genotype strains in Egypt (Hagag et al., 2022). The findings of this study support the fact that HPAI H5N8 viruses have replaced formerly endemic HPAI H5N1 viruses of clade 2.2.1.2 in both the backyard and commercial farming sectors since 2016 (Amer et al., 2021). The A/quail/FAO-S98 and HPAI H5N8 viruses from Egypt carry amino acids within the receptor-binding domain (RBD) of their HA indicating binding preference to α2,3- sialic acid receptors “avian receptor” (Cai et al., 2012; Mair et al., 2014). The likelihood of human infection with influenza A (H5N8) viruses has been limited worldwide. Only seven farm workers were infected with HPAI H5N8 in the Astrakhan Oblast region of the Russian Federation (Ding et al., 2022). To date, no human infections caused by HPAI H5N8 virus have been reported in Egypt. The globally limited numbers of human infections of HPAI H5N8 may be attributed to the presence of residues at positions Q222 and G224 in the HA, which confer avian-type receptor-binding specificity (sialic acid linked to the penultimate galactose residue by an α2,3- linkage). In contrast, all analysed HA sequences of HPAI H5N8 from Egypt did not express residues L222 and S224, which confer human-type receptor-binding specificity (sialic acid linked to the penultimate galactose residue by an α2,6- linkage) (Rogers and Paulson, 1983; Stevens et al., 2006; Connor et al., 1994). Moreover, all published HPAI H5N8 strains do not contain PB2–627K, which confers efficient replication in mammals (Hatta et al., 2001). In addition, the quail strain carrys amino acids indicating sensitivity to amantadine (Govorkova et al., 2013) and neuraminidase inhibitors (Choi et al., 2017; Jeong et al., 2020; Bialy and Shelton, 2020; Svyatchenko et al., 2021) in its M2 and NA coding proteins respectively. Surveillance and genetic monitoring (Olsen et al., 2006) help us to predict which strains and subtypes represent the greatest risk, and further investigations of strains with potential human transmission are needed. A crucial step in this approach is to identify phenotypic markers derived from sequence data that are relevant for determining risk (Yamada et al., 2006; Dugan et al., 2012; Pepin et al., 2010).

In conclusion, the genome sequence of the avian influenza virus isolated from A/quail/FAO-S98 evealed that the strain is the HPAI H5N8 strain and belongs to clade 2.3.4.4b with close relatedness to HPAI H5N8 previously isolated from Egypt. The quail strain possesses several mutations in different genes that have the potential to increase virulence in mammals. The detection of HPAI H5N8 in quail supports the continuing spread of this virus to different species in Egypt. This calls for continuous surveillance, early diagnosis, and genetic characterization of detected viruses to monitor the emergence of new influenza virus strains or novel phenotypic markers, which in turn help mitigate the risk to poultry populations in Egypt.

## Declaration of generative AI in scientific writing

The authors declare no usage of any Articfical Intellegnce tool in the writing of the manuscript.

## Research funding

The study was funded by internal core funds from the Animal Health Research Institute, Ministry of Agriculture, Giza, Egypt. This work is partially supported by Carl Trygger Stiftelse (grant number CTS 23:2747) and Åke Wibergs Stiftelse (grant number M22-0074) to M.M.N.

## CRediT authorship contribution statement

**Mohamed H. Elhusseiny:** Writing – original draft, Validation, Methodology, Formal analysis, Data curation, Conceptualization. **Moataz M. Elsayed:** Methodology, Conceptualization. **Wesam H. Mady:** Writing – original draft, Methodology, Data curation. **Osama Mahana:** Methodology. **Neveen R. Bakry:** Methodology. **Ola Abdelaziz:** Methodology. **Abdel-Sattar Arafa:** Supervision, Investigation. **Momtaz A. Shahein:** Validation. **Samah Eid:** Resources. **Mahmoud M. Naguib:** Writing – review & editing, Visualization, Validation, Supervision, Software, Investigation, Funding acquisition, Formal analysis.

## Declaration of competing interest

The authors declare that there are no conflicts of interest.

## Data Availability

The sequences generated in this study are publicly available at GenBank under accession number JX438661 MH893737 MF037848 KR732532 CY020649 EU146856 KR732442 KR732472.
